# *Lactobacillus paracasei* GMNL-32 exerts a therapeutic effect on cardiac abnormalities in NZB/W F1 mice

**DOI:** 10.1371/journal.pone.0185098

**Published:** 2017-09-21

**Authors:** Wei-Syun Hu, Peramaiyan Rajendran, Bor-Show Tzang, Yu-Lan Yeh, Chia-Yao Shen, Ray-Jade Chen, Tsung-Jung Ho, Viswanadha Vijaya Padma, Yi-Hsing Chen, Chih-Yang Huang

**Affiliations:** 1 School of Medicine, College of Medicine, China Medical University, Taichung, Taiwan, ROC; 2 Division of Cardiovascular Medicine, Department of Medicine, China Medical University Hospital, Taichung, Taiwan, ROC; 3 Graduate Institute of Basic Medical Science, China Medical University, Taichung, Taiwan, ROC; 4 Institute of Biochemistry and Biotechnology, Chung Shan Medical University, Taichung, Taiwan, ROC; 5 Department of pathology, Changhua Christian Hospital, Changhua, Taiwan, ROC; 6 Jen-Teh Junior College of Medicine, Nursing and Management, Miaoli, Taiwan, ROC; 7 Department of Nursing, Mei Ho University, Pingtung, Taiwan, ROC; 8 Department of Surgery, School of Medicine, College of Medicine, Taipei Medical University, Taipei, Taiwan, ROC; 9 Graduate Institute of Chinese Medical Science, China Medical University, Taichung, Taiwan, ROC; 10 Department of Biotechnology, Bharathiar University, Coimbatore, India; 11 Research and Development Department, GenMont Biotech Incorporation, Tainan, Taiwan, ROC; 12 Department of Health and Nutrition Biotechnology, Asia University, Taichung, Taiwan, ROC; Institute of Biochemistry and Biotechnology, TAIWAN

## Abstract

Systemic lupus erythematosus (SLE) is a disease that mostly affects women. Accelerated atherosclerosis is a high-risk factor associated with SLE patients. SLE associated with cardiovascular disease is one of the most important causes of death. In this study, we demonstrated that *Lactobacillus paracasei* GMNL-32 (GMNL-32), a probiotic species, exhibits anti-fibrosis and anti-apoptotic effects on the cardiac tissue of NZB/WF1 mice. Female NZB/W F1 mice, a well-known and commonly used lupus-prone mouse strain, were treated with or without GMNL-32 administration for 12 weeks. Oral administration of GMNL-32 to NZB/WF1 mice significantly increased the ventricular thickness when compared to that of NZB/WF1 mice. Administration of GMNL-32 significantly attenuated the cardiac cell apoptosis that was observed in exacerbate levels in the control NZB/WF1 mice. Further, the cellular morphology that was slightly distorted in the NZB/WF1 was effectively alleviated in the treatment group mice. In addition, GMNL-32 reduced the level of Fas death receptor-related pathway of apoptosis signaling and enhanced anti-apoptotic proteins. These results indicate that GMNL-32 exhibit an effective protective effect on cardiac cells of SLE mice. Thus, GMNL-32 may be a potential therapeutic strategy against SLE associated arthrosclerosis.

## Introduction

Systemic lupus erythematosus (SLE) is an autoimmune disorder that only affects women. SLE patients may show modulations in cardiac tissue including the pericardium, coronary arteries, valves, myocardium, and conduction system [1]. The occurrence of Coronary heart diseases in SLE can be due to many pathophysiologic mechanisms, including arteritis, thrombosis, abnormal coronary flow, embolization, and atherosclerosis [2]. Patients with SLE have a high rate of coronary heart disease [3], generally as pericarditis and myocarditis and Cardiovascular disease is considered to be the primary cause of morbidity and mortality in SLE [4]. It has been reported that women of 44–50 years of age have a 50-fold increased risk of myocardial infarction [5].

Autoantibodies, including anti-phospholipid antibodies and anti-endothelial antibodies are known to inflict cardiac damages [4,6–9]. In SLE patients apoptosis has been firmly related with different autoantibodies such as anti-phospholipids and anti-oxidized low-density lipoprotein antibodies and the engagement of these autoantibodies with self-tissue is considered to enact the supplement framework, cell-interceded cytotoxicity, and cardiomyocyte apoptosis [10,11]. For this reason, restraint of cardiovascular apoptosis is recommended to improve autoantibody-incited heart injuries in SLE patients.

Several studies have proven that probiotics detain the crucial not only to health and a stronger immune system but also for the treatment of metabolic diseases [12–14]. *Lactobacilli* comprise various probiotic strains that exert beneficial effects through anti-inflammatory actions, intestinal barrier stabilization, and possible attenuation of liver disorders[15]. Administering live probiotics in immunocompromised patients is a risky affair. While healthy individuals can tolerate the presence of effects of probiotics in their gastrointestinal system, patients with modulated immune response may be at a risk of infection [16]. Many studies have shown that heat-killed probiotics have greater beneficial effects [17–22].

Recently probiotics have been shown to have the ability to affect metabolic fat and improve the immune response and stress resistance [23,24]. By decreasing absorption and inflammatory status, *Lactobacillus gasseri* SBT2055 can able to decrease body weight, obesity, and adiposity in obese adults who consumed fermented milk with this bacterium for 12 weeks [25]. To-shimitsu et al. reported that treatment of KKAy mice with the *L*. *plantarum* strain OLL2712 was effective in alleviating metabolic disorders by suppressing chronic inflammation in the KKAy mice [26]. Likewise, probiotic bacteria may impact various components of atherogenesis, e.g., *Lactobacilli* have been appeared to bring down blood cholesterol levels in both rodents and in humans [27,28], by balancing cholesterol re-absorption from the gut through its effects on the bile-digestion system. Very few studies have explored probiotic interventions on atherosclerosis advancements in animal models. Portugal LR *et al*., [28] treated the Apoe−/− mouse model with *L*. *delbruecki* but observed to no significant changes in the lesion size. However, the bacterium caused no change in blood cholesterol levels, and the mice were colonized with *L*. *delbruecki* for approximately 4 to 10 weeks of age, which can be considered moderately early in disease progression [29].

Feeding heat-killed *L*. *acidophilus* and *L*. *casei* had showed ameliorative effect on *Candida albicans*-colonized immune-deficient mice, although the immunomodulatory effects of these candidate probiotic bacteria showed that differences exist between them [30]. Wang *et*.*al*, shown that *L*. *paracasei* ssp *paracasei* F19 (F19) block diet-induced obesity in mice [31]. *L*. *paracasei* has also been known to exhibit cardio-protection in murine models. Our previous study shows that heat killed *L*. *paracasei* effectively improves the cardiac function and inhibits the myocardial apoptosis in high-fat-diet fed hamsters and rat models. They are also effective in ameliorating the cardiac as well as hepatic fibrosis effects associated with high calorie-diet feeding and are also known to strongly suppress the inflammation mediators [32–34]. In the present study, we therefore investigate the impacts of *L*. *paracasei* GMNL-32 strain on the hearts of NZB/W F1 mice, with respect to modulation in apoptosis and survival signaling pathways.

## Materials and methods

All the protocols of animal experiments were reviewed and approved by the Institutional Review Board (IRB), and the Animal Care and Use Advisory Group of the China Medical University, Taichung, and Republic of China.

### Preparation of *Lactobacillus paracasei* GMNL-32 (GMNL-32)

GMNL-32 was stored in the Bioresource Collection and Research Center, Taiwan (BCRC 910220), and the China Center for Type Culture Collection, China (M204012). GMNL-32 was provided by Gen Mont Biotech Inc., Tainan, Taiwan. GMNL-32 was diluted with PBS and 10^9^ CFU/mouse per day by oral gavage.

### Mice and diets

Female NZB/W F1 mice-an outstanding and commonly used lupus-prone mouse strain–was obtained from the animal center, National Taiwan University, Taiwan and housed in an animal room at 22 ±2°C with a 12/12 h light-dim cycle under supervision of the Institutional Animal Care. The diseased state of mice was determined by observing proteinuria with Albustix test strips every other week from the age of 12 weeks as previously described [17]. Animals were separated randomly into two groups (n = 10 each). Group I was the control group mice that were provided with oral saline solution and group II was orally administered with GMNL-32 (1 × 10^9^CFU/mL per day). From the 8^th^ week, SLE-developing mice (group II) were treated orally with GMNL-32, continuously up to the 20^th^ week. After 3 weeks treatment, all rats were sacrificed by decapitation under terminal anaesthesia and hearts were collected. Heart tissues of the mice were obtained and stored at– 80°C until use.

### Hematoxylin and eosin staining

The hearts of the test mice were removed, fixed in formalin, dehydrated by alcohol gradient (100%, 95%, and 75%) and were embedded in paraffin wax. The tissue squares were cut into 0.2 μm-thick slices and deparaffinized by submersion in xylene. The slices were stained with hematoxylin and eosin (H&E) and washed with water. Each slide was dried using an alcohol gradient and rinsed twice with xylene. Photomicrographs were acquired using a Zeiss Axiophot magnifying lens (Carl Zeiss Microscopy, Thornwood, NY, USA).

### DAPI staining and TUNEL assay

For the terminal deoxynucleotidyltransferase2'-Deoxyuridine,5'-Triphosphate (dUTP)- nick end labeling (TUNEL) assay, the sections were treated with proteinase K, washed in PBS, incubated with permeabilization solution, followed by blocking buffer, and washed two times with PBS. The sections were then treated for 60 min at 37°C with terminal deoxynucleotidyl transferase and fluorescein isothiocyanate-dUTP from an apoptosis detection kit (Roche Applied Science, Indianapolis, IN, USA). Under light (excitation wavelength of 460 nm) and detection in the range of 515–565 nm, TUNEL-positive cells (divided DNA) were identified as brilliant green. The tissue sections were stained with 0.1 μg/mL 4, 6-diamidino-2-phenylindole (DAPI) for 5 min, and the cell nuclei were identified by UV light microscopy at 454 nm. Photomicrographs were acquired using a Zeiss Axio photo magnifying instrument.

### Tissue protein extraction

Cardiovascular tissue concentrates of the mice were acquired by homogenizing the heart (100 mg/mL). The homogenates were put on ice and then centrifuged at 12,000 g for 40 min. The proteins in the supernatants were collected and stored at -80°C for further analyses.

### Western blot

Protein concentrations of the heart tissue concentrates were determined by Lowry's protein assay. Proteins were separated by 12% SDS polyacrylamide gel electrophoresis (SDS-PAGE) with a consistent supply of 75 and were then transferred to a poly vinylidene difluoride (GE Healthcare Life Sciences, Pittsburgh, PA, USA) membrane using a 50 V current for 3h. The membrane was treated with 3% bovine serum albumin (BSA) in Tris-buffered saline solution followed by incubation with primary antibodies to particular proteins (Santa Cruz Biotechnology, Santa Cruz, CA, USA). Horseradish peroxidase-labeled secondary antibodies were used for detection, and pictures were taken with Fujifilm LAS-3000 (GE Health Mind Life Sciences).

### Immunofluorescence

After the fixation, rehydration, and blocking of the slides, the primary antibody MMP9 was added for detection of the lysosomes in heart sections. After that, a goat anti-rabbit IgG secondary antibody, Alexa Fluor® 488 conjugate (A-11008, Thermo Fisher, USA), was used to detect the bound MMP9 primary antibody. The cell nuclei were stained with DAPI as the last step before mounting the sections. The pictures were acquired using an Immunofluorescence microscope (CKX53, Olympus, Tokyo, Japan).

### Masson’s trichrome staining

Masson's trichrome recoloring the heart tissue from each gathering was put away in 10% formalin for 2 weeks, got dried out utilizing a alcohol gradient (75%, 85%, 90%, and 100% liquor, 5 min each) and inserted in paraffin wax. Paraffin sections that were 0.2 μm thick were then cut from these paraffin-installed tissue squares. The tissue areas were de-paraffinized by dipping in xylene (3 times, 5 min each) and rehydrated utilizing an alcohol gradient (100%, 90%, 85%, and 75% ethanol, 5 min each). Tissue sections were then stained utilizing Masson's trichrome stain to examine heart morphologic and fibrotic changes; blue staining indicated collagen thickening. The results were acquired using an OLYMPUS microscope.

### Statistical analysis

The results indicated are the means ± SD of three independent trials. Statistical analysis was performed by one-way analysis of variance. For comparison between two groups, Student's t-test was used.

## Results

### GMNL-32 induces weight gain in lupus-prone mice

GMNL-32 nourishing adequately instigated weight pick up in lupus inclined mice groups. The rate of weight pick up did not altogether contrast between NZB/W F1 control and GMNL-32 treated mice groups, but the whole heart weight was significantly increased in GMNL-32 treated animals compared to the NZB/W F1 mice. Left ventricular weight (LVW) and the proportions of entire heart weight to tibia length (WHW/Tibia) and left ventricular weight to tibia length (LVW/Tibia) in the GMNL-32 groups did not show much difference when compared to the SLE control group (Table 1).

**Table 1:** Effects of GMNL-32 body and Heart weight on NZB/W F1 mice.

Particulars	SLE (n = 10)	SLE+GMNL-32 (n = 10)
**No of Animals**	10	10
**Body Weight (BW),g**	33.44±5.03	45.01±1.83***
**Whole Heart (WHW),g**	0.128±0.021	0.155±0.013*
**Left Ventricular weight (LVW),g**	0.092±0.016	0.107±0.006*
**WHW/Tibia g m/m (×10^4^)**	73.58±0.0014	72.06±0.0016**
**LVW/Tibia, g/mm (×10^4^)**	53.26±0.0032	52.31±0.0016**

Values are Mean ± S.E., n = 10

***p<0.001

**p<0.01

* *p*< 0.05 represent significance when compared to NZB/W F1 mice group.

### GMNL-32 strain increases the ventricular wall thickness

**Fig 1:**
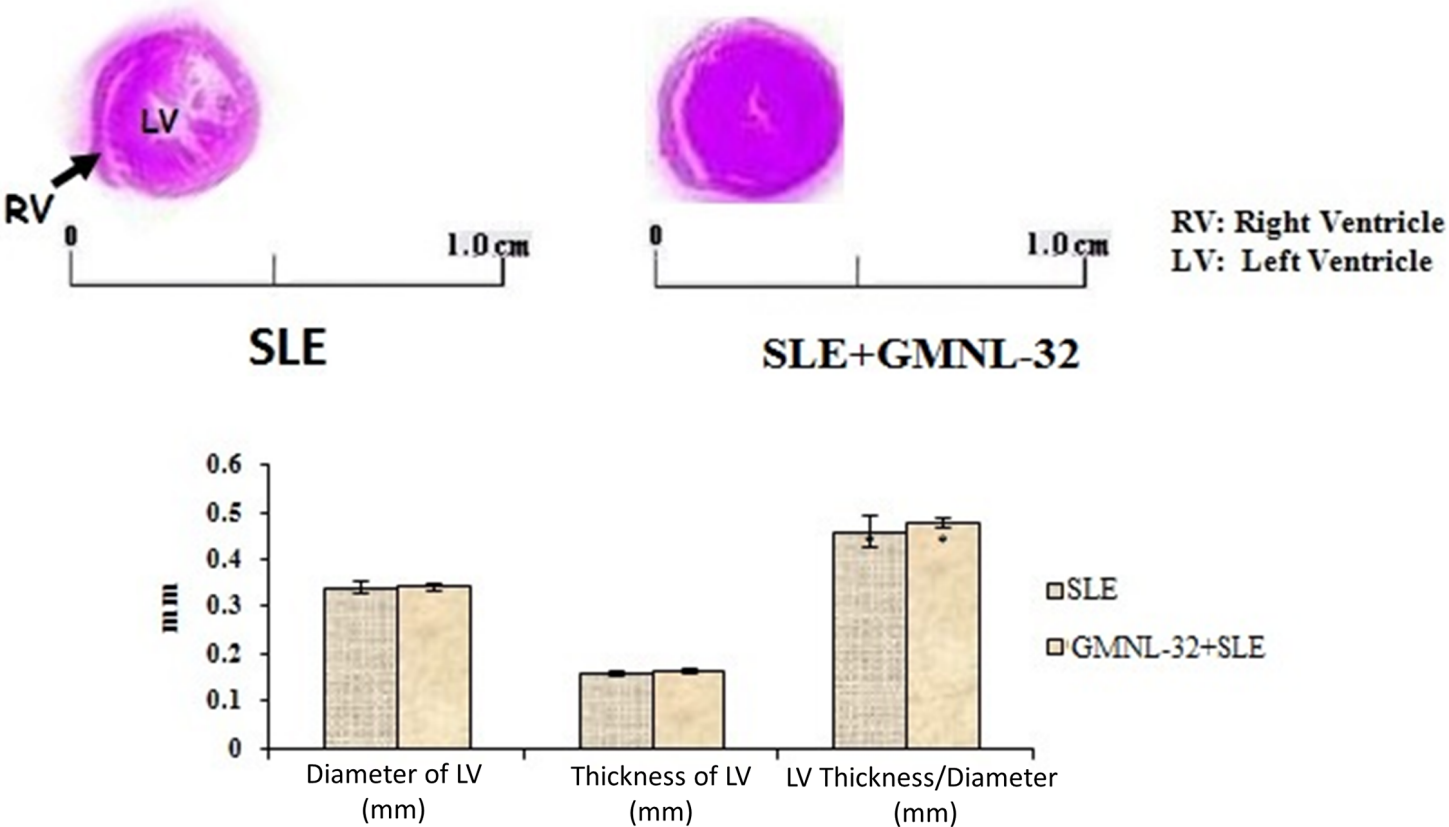
Effect of GMNL-32 on Ventricular wall thickness. A). left ventricular wall thickness measured by imagej software, B). left ventricular wall thickness significance. Values are Mean ± S.E., n = 10. * p< 0.05 represents significance when compared to NZB/W F1 mice group.

Histopathological tomography analyses of whole heart tissues were performed using H&E staining (Fig 1). Images were viewed under a microscope. Fig 1A shows that the left ventricular wall thickness of the control SLE mice is smaller than that of the SLE + GMNL-32 mice, and the difference is statistically significant (Fig 1B). From the results of the above cross-section view, SLE + GMNL-32 tissue wall thickness is greater than the wall thickness of the SLE group.

### Cardiac histopathological changes and Fas Death receptor-related components in NZB/W F1 mice treated with GMNL-32

**Fig 2:**
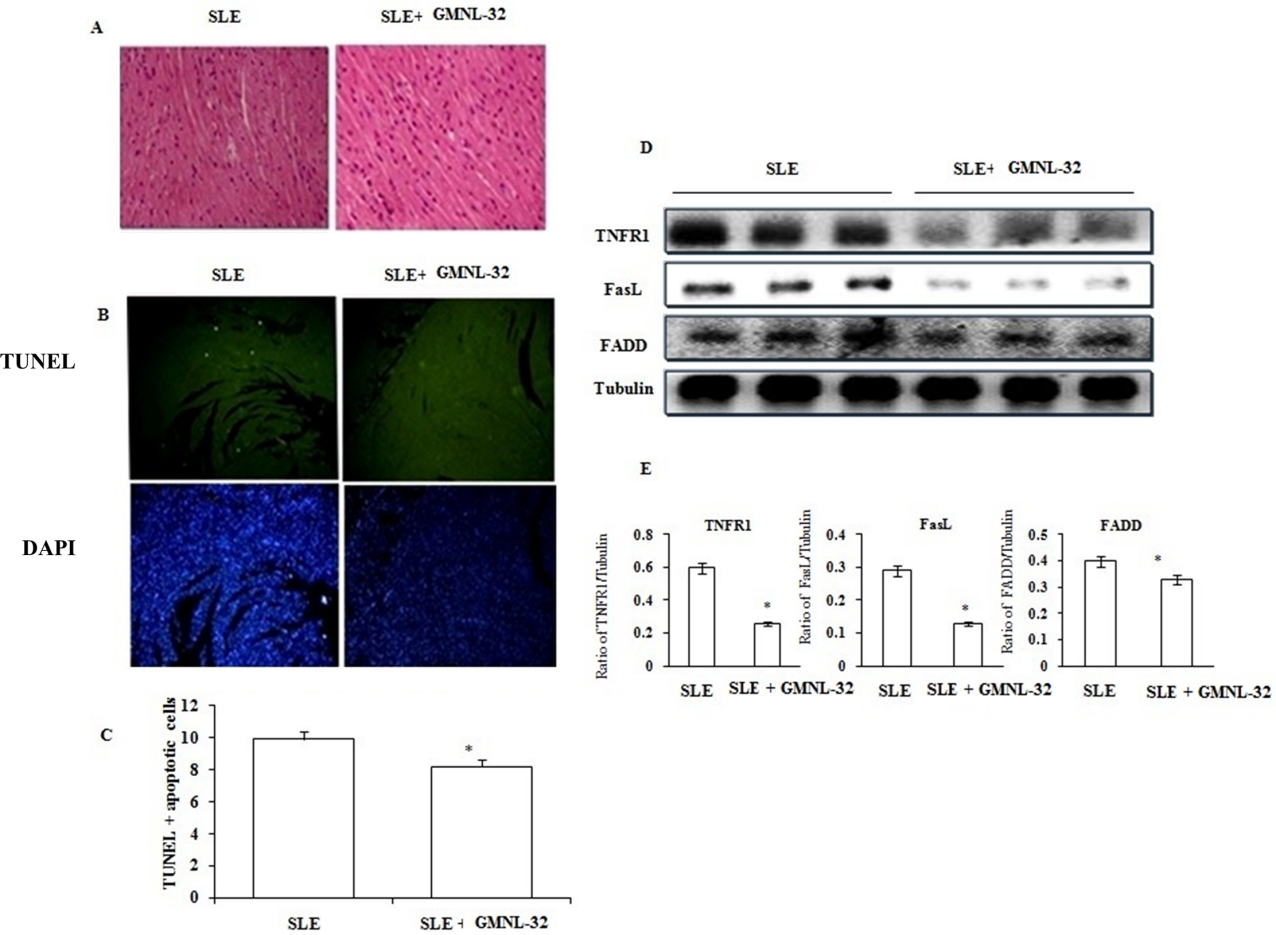
Effect of GMNL-32 on hematoxylin staining and Fas-induced apoptosis pathway signaling. A). Histopathological analysis of tissue section slides stained by hematoxylin and eosin staining B). Recolored apoptotic cells of cardiovascular areas with TUNEL test in NZB/W F1 mice encouraged with supplementation. C).The rates of apoptotic cells were computed. The pictures of myocardial design were amplified 100 times. Protein products of TNF-R1, Fas, and FADD in the left ventricles of hearts from NZB/W F1 mice encouraged with GMNL-32 were measured by Western blotting investigation. α-tubulin filled in as an inward control. D).The relative protein evaluation of TNF-R1, Fas, and FADD on the premise of fold change. Bars exhibit the rate of TUNEL-positive cells relative to add up to cells (10 mice X10 scope field number in each gathering) and show mean esteems (SD ± * p< 0.05 represents significance when compared to NZB/W F1 mice group.

To explore whether the myocardial design and cardiovascular apoptosis were expanded in the hearts of NZB/W F1 mice treated with GMNL-32, a histopathological analysis of the left cardiac tissue was performed with hematoxylin and eosin staining and TUNEL assay. NZB/W F1 mice group exhibited a more abnormal architecture. In contrast, a less abnormal architecture was seen in the NZB/W F1 mice + GMNL-32 group compared to the NZB/W F1 mice group (Fig 2A). Additionally, NZB/W F1 hearts stained by TUNEL assay showed increased TUNEL-positive cardiac cells in the NZB/W F1 mice, whereas decreased TUNEL-positive nuclei were observed in the GMNL-32 group (Fig 2B). The average percentages of TUNEL-positive cardiac nuclei in the GMNL-32 groups were 9.91± 1.81 and 8.23± 0.93, (Fig 2C). To determine the Fas death receptor involved apoptosis in the hearts of NZB/WF1 mice, Western blotting was performed. The levels of TNF-R1, Fas receptors, and FADDs in the hearts of NZB/W F1 mice group were significantly increased (Fig 2D). In contrast, TNF-R1, Fas receptors, and FADDs were all found to be reduced in the GMNL-32 administered group (Fig 2D). The fold change in the ratios of the protein products of TNF-R1, Fas receptors, and FADDs relative to the internal control are shown in Fig 2E.

### Changes in cell survival components in the hearts of NZB/W F1 mice treated with GMNL-32

**Fig 3:**
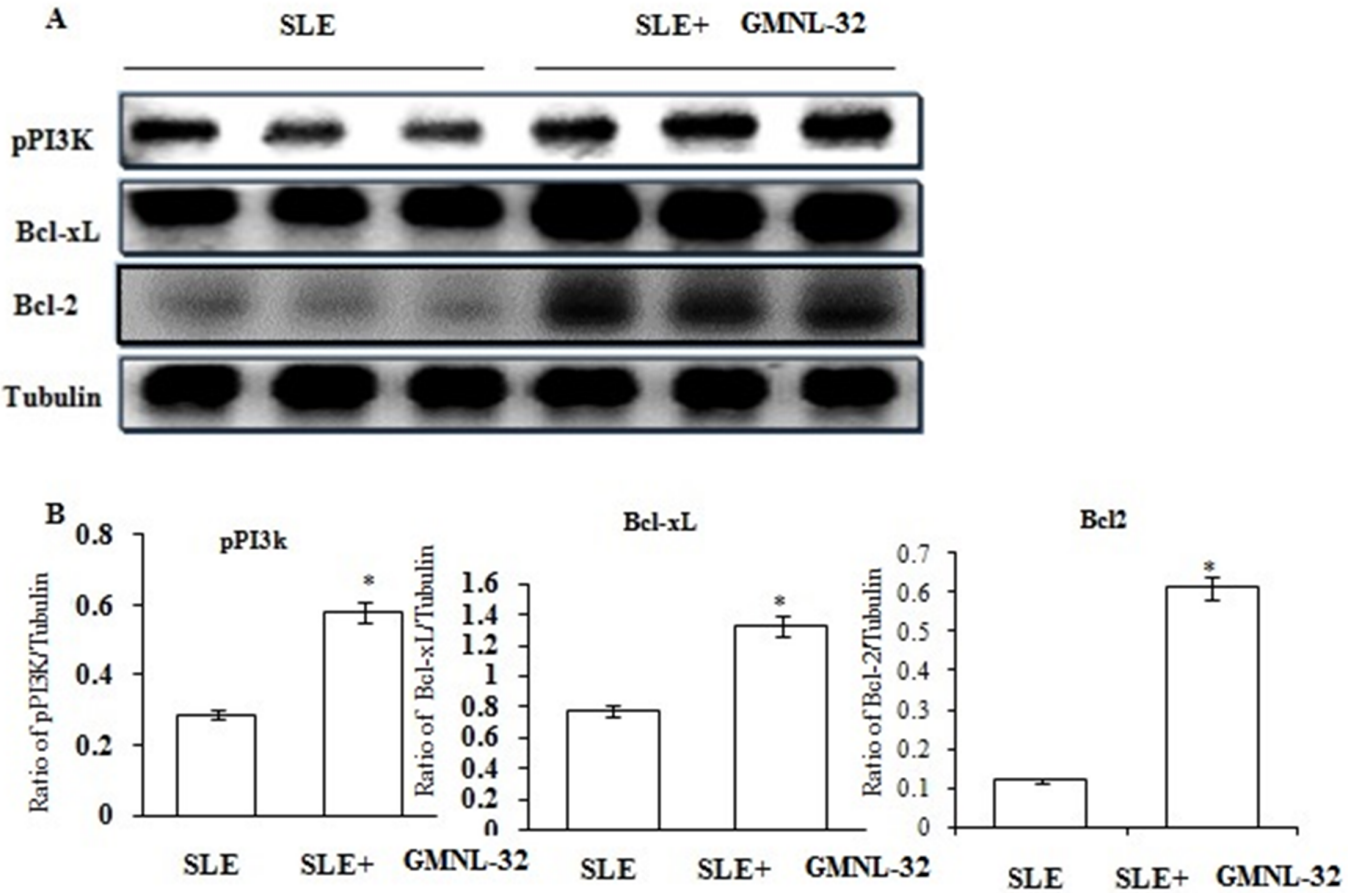
Effect of GMNL-32 on survival signaling proteins. A). Protein products of PI3K, Bcl-xl, and Bcl2 in the hearts from NZB/W F1 mice treated with GMNL-32 were measured by Western blotting analysis. α-tubulin filled in as an inside control. B). the relative protein quantification of PI3K, Bcl-xl, and Bcl2 on the basis of α –tubulin. * p< 0.05 represents significance when compared to NZB/W F1 mice group.

To examine the variety of cardiovascular survival protein segments in the hearts of NZB/W F1 mice, the levels of PI3K, Bcl-xl, and Bcl2 were examined as shown in Fig 3. The levels of PI3K, Bcl-xl, and Bcl2 (Fig 3A) were significantly decreased in the hearts from the NZB/W F1 mice. In contrast, these protein levels were significantly increased in the NZB/W F1 + GMNL-32 group. The relative protein quantification expressed as fold change is show in Fig 3B.

### Change in cardiac fibrosis in the hearts of NZB/W F1 mice treated with GMNL-32

**Fig 4:**
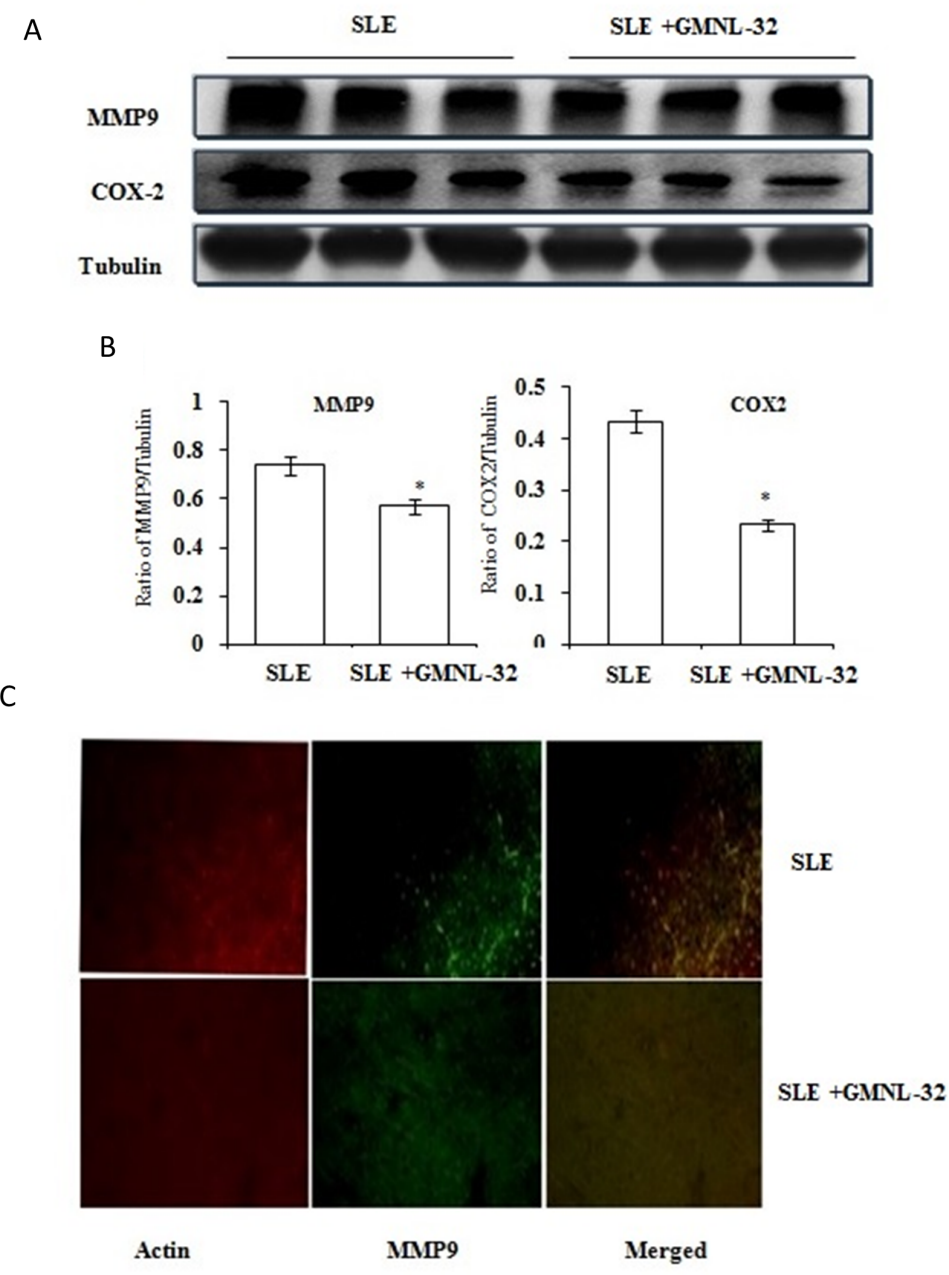
Effect of GMNL-32 on MMP9 and COX2 levels. A). Protein products of MMP9 and COX2 in the hearts from NZB/W F1 mice treated with GMNL-32 were measured by Western blotting analysis. α-tubulin filled in as an inward control. B). the relative protein measurement of MMP9 and COX2 on the basis of α -tubulin. * p< 0.05 represents significance when compared to NZB/W F1 mice group.(B) Panel A shows the immunofluorescence images of MMP-2 in NZB/W F1 mice and GMNL-32 treatment.

In the hearts of NZB/W F1 Mice, fibrosis proteins MMP-9 and Cox2 were examined by Western blotting (Fig 4). The protein levels of MMP-9 and Cox2 in NZB/W F1 mice were significantly increased, whereas in NZB/W F1 mice treated with GMNL-32, the levels of these proteins were decreased (Fig 4A). Transformation of cardiac fibroblasts into myofibroblasts is a critical event in the initiation of myocardial fibrosis. Further to detect the effects of GMNL-32 on the events associated with cardiac remodeling in NZB/W F1 mice, the level of MMP9 was determined by immune-fluorescence staining (Fig 4C). Elevated levels of MMP9 and CoX2 observed in NZB/W F1 mice group were found to be downregulated in the GMNL-32 treated group.

### Change in cardiac collagen accumulation in the hearts of NZB/W F1 Mice treated with GMNL-32

**Fig 5:**
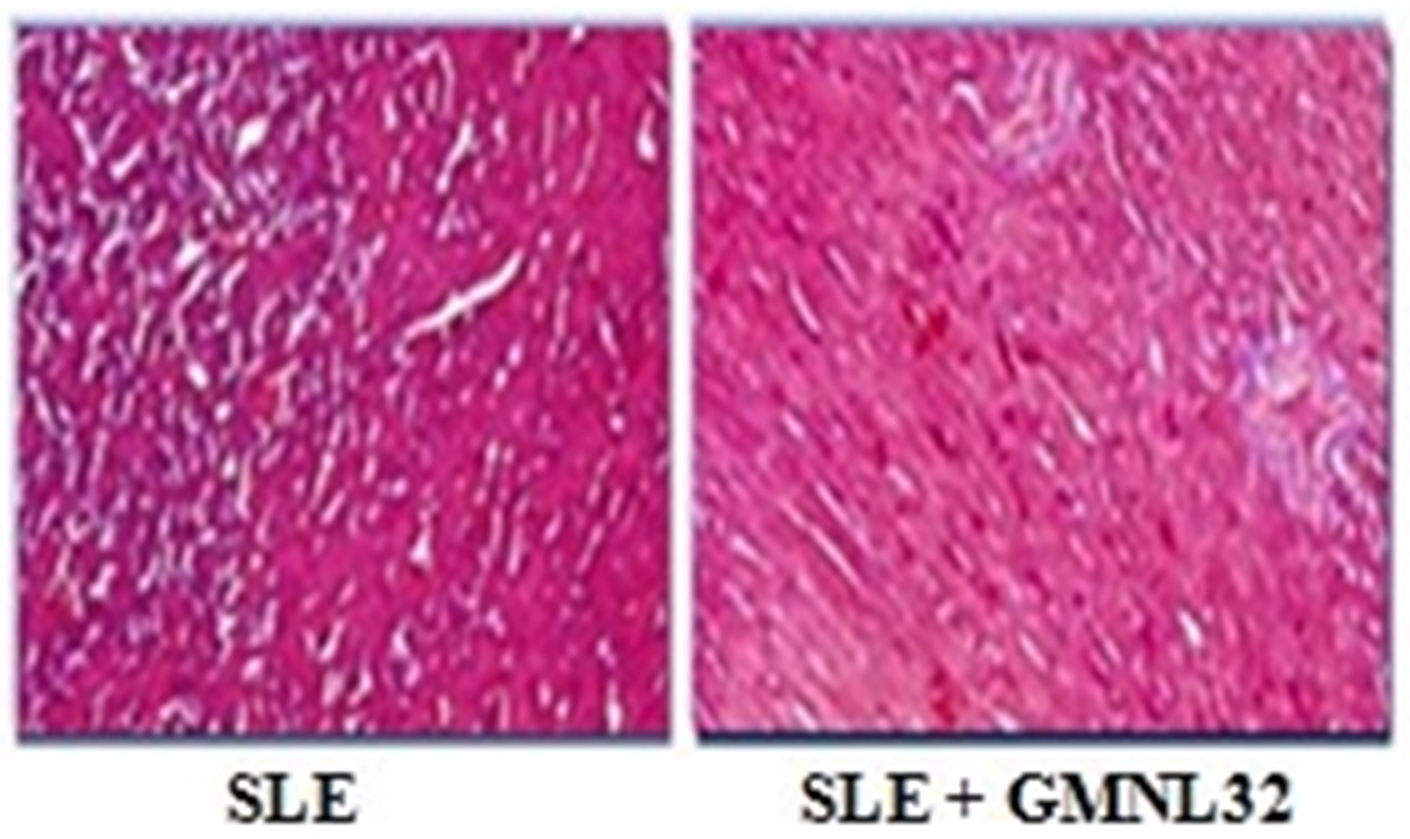
Effect of GMNL-32 on collagen accumulation. Masson trichrome staining shows fibrosis of hearts from NZB/W F1 mice treated with GMNL-32.

Cardiac tissue sections from the NZB/W F1 control mice and GMNL-32 treated mice NZB/W F1 mice were stained by Masson’s trichrome staining and the results showed that cardiac cellular arrangement was disordered, with noticeably high collagen accumulation (blue color) (Fig 5). Supplementation with GMNL-32 probiotics however, rescued the heart tissue as evident from the reduction in the collagen accumulation.

## Discussion

Systemic lupus erythematosus is an immune system inflammatory illness influencing different organs and is characterized by aggressive inflammation and reduction in the life expectancy. Hereditary, hormonal, and natural elements creating chronic inflammation are considered as causes of SLE. In its course, the illness affects different organs, including the lungs, heart, kidneys, mind, fringe nerves and skin [35,36]. SLE has a female dominance (9:1) and has a prevalence of 15–50/100,000 people, with side effects generally showing up between the second and third decades of life [37]. Early mortality in SLE is ascribed to either renal failure because of uncontrolled disease or high vulnerability to infections, while late mortality is because of cardiac complications and hematological malignancies [38,39]. The effect of contaminations and high infection impact on mortality has decreased drastically in the most recent decades. Not with standing, cardiovascular disease (CVD) has emerged as an essential contributor to mortality [40], as has been proven by the high occurrence of myocardial localized necrosis in young women with SLE [40]. Our results reveal that there was a significant increase in body weight in NZB/W F1 mice groups treated with GMNL-32 however there was no significant change observed in the heart weight or the left ventricle weight. Body Weight loss is a well-known effect in patients with active SLE however weight gain may also be noticed in the patients due to corticosteroid treatment provided against inflammation[9]. In our SLE model, GMNL-32-treatment caused weight gain may reflect the reduction in the pathological effects of SLE either in the form of reduced autoimmunity or due to reduction in the mediators of inflammatory. Observation on increased ventricular mass in SLE patients has been associated with hypertension and modulation in LV are not observed in normotensive patients [41]. In our results there was no significant difference observed in the whole heart weight or the left ventricle weight.

CVD in SLE is thought to occur as the consequence of increase in the conventional risk factors of CVD along with disease progression. Concerning that, a systemic investigation and meta-analysis on 17,187 SLE patients for a subsequent time of eight years demonstrated that CVD events happened in 25.4% of the patients [36]. The pathological variations in the heart include histopathological changes, cellular apoptosis, and elevated fibrotic lesions in the heart tissue. In this study, *L*. *paracasei* strain GMNL-32 diminished apoptosis in the heart tissues of NZB/W F1 mice and potentially enhanced cardiac function. The finding gives some convincing insight into the probiotic potential of *L*. *paracasei* strain GMNL-32 and reveals the protective effect of GMNL-32 against cardiovascular apoptosis related in SLE.

Despite the fact that *L*. *paracasei* has been considered to be effective against various pathological conditions little is known about how the *L*. *paracasei* strain balances the immune system, atopic ailments and autoimmunity improvement. Presently, only limited studies are available characterizing the impacts of probiotics in murine or human models of atopy and autoimmunity. Therefore, it is essential to investigate the impact of probiotics in different trial and clinical atopic and immune system disease models [42–48]. The *L*. *paracasei* strain has been shown to deliver ideal probiotic impacts [29]. Past reviews have demonstrated that *L*. *paracasei* diminishes reactive oxygen species to protect against hepatocyte injury [33] and attenuates the production of pro-inflammatory cytokines [49,50]. The protective effects of *L*. *paracasei* in decreasing serum lipid, lipid oxidation and keeping the arrangement of the caspase-9 apoptosome [51] were previosuly reported. Another report demonstrated that *L*. *paracasei* lessens cardiovascular break down and apoptosis incited by Ca^2+^by means of decreasing MAPK and apoptotic signaling segments [52,53]. Altogether, decreased apoptotsis positive cells were distinguished in heart tissues of the GMNL-32 group compared to the NZB/W F1 mice. Both Fas-and mitochondria-dependent mediators such as TNF-R, Fas ligand, and FADD, were altogether diminished in heart tissues of the GMNL-32 treated mice groups when compared to the NZB/W F1 mice group. Additionally, reduced fibrosis and diminished fibrotic signaling molecules, such as MMP-9 and COX2, were seen in the heart tissues of the GMNL-32 group compared to the NZB/W F1 mice. Matrix metalloproteinase-9 (MMP-9) has been hypothesized with the pathogenesis of immune system infections including SLE. Different examinations have detailed that hoisted MMP-9 movement assumes urgent part being developed of SLE in both human and lupus-inclined mice. COX-2 is likewise known to assume crucial parts being developed of incendiary ailments and related with the pathogenesis of SLE. COX-2 and MMP-9 expression is managed by inflammation and a useful connection between COX-2 action and MMP-9 generation has been portrayed in various cell sorts, including endothelial cells, proposing COX-2 and related MMP-9 as vital helpful focuses for heart anomaly hindrance. Late reports have highlighted the mutual pathology of cardiovascular diseases and SLE, both of which speak to incendiary issue. A few reports have additionally given truly necessary understanding into the malicious effect that select treatments (counting cyclo-oxygenase-2-specific inhibitors) may have as far as the danger of cardiovascular illness in SLE [54]. Further MMP9 levels can be correlated with the level of collagen accumulation and risk of cardiac remodeling after cardiac injury [55]. Our results clearly show that GMNL-32 provide cardio–protective effects in SLE diminishing the pathways of cardiac apoptosis. The initiation of the PI3K/Akt pro-survival pathway plays an important role in cardio protection [56]. In the present review, the levels of survival protein expression were too low in NZB/W F1 mice, whereas *L*. *paracasei* strain GMNL-32 treatment in mice caused a change in the expression of these ACE and anti-apoptotic proteins, particularly Bcl-2, Bcl-xl. The PI3K/Akt flagging pathway may be involved in the regulation of the expression of antiapoptotic proteins in *L*. *paracasei* strain GMNL-32-treated mice. *L*. *paracasei* has been known to be effective in the treatment of organ disorders, particularly, against liver injury, and in cardio protection [33]. Nonetheless, *L*. *paracasei* GMNL-32 uncovers the effects on cardiovascular tissue by diminishing the histopathological changes, Fas-subordinate apoptosis, fibrosis, and fibrotic aggregations in the heart tissues of NZB/W F1 mice and also by expanding cardiovascular survival flagging segments in NZB/W F1 mice. These discoveries may provide significant information for understanding the cardiovascular protective function of *L*. *paracasei* GMNL-32 and suggest the capability of GMNL-32 in treating SLE patients with CVD.

## Financial disclosure

This work is supported by Taiwan Ministry of Health and Welfare Clinical Trial and Research Center of Excellence (MOHW105-TDU-B-212-133019) and GenMont Biotech Incorporation, Taiwan. (1044EI). The funding organization provided support in the form of salaries for authors [Y-H.C] and research materials (*L*. *paracasei* GMNL-32), but did not have any additional role in the study design, data collection and analysis, decision to publish, or preparation of the manuscript. The specific roles of these authors are articulated in the ‘author contributions’ section.
